# The role of genomics and genetics in pulmonary arterial hypertension

**DOI:** 10.21542/gcsp.2020.13

**Published:** 2020-04-30

**Authors:** Emilia M. Swietlik, Stefan Gräf, Nicholas W. Morrell

**Affiliations:** 1 Department of Medicine, University of Cambridge, Cambridge Biomedical Campus, Cambridge, United Kingdom; 2 Addenbrooke’s Hospital NHS Foundation Trust, Cambridge Biomedical Campus, Cambridge, United Kingdom; 3 Royal Papworth Hospital NHS Foundation Trust, Cambridge Biomedical Campus, Cambridge, United Kingdom; 4 Department of Haematology, University of Cambridge, Cambridge Biomedical Campus, Cambridge, United Kingdom; 5 NIHR BioResource for Translational Research, Cambridge Biomedical Campus, Cambridge, United Kingdom

## Introduction

Although pulmonary hypertension (PH) had been recognised for centuries, it was not until the invention of cardiac catheterisation in the 1950s that enabled an accurate clinical diagnosis^1^. The discovery of heterozygous germline mutations in *BMPR2*, the gene encoding bone morphogenetic protein receptor type 2, in patients with familial and idiopathic forms of pulmonary arterial hypertension (PAH) was another breakthrough in understanding the disease and initiated a new era in care of patients with this condition^2^. It has been reported that around 70–80% of familial PAH and 10–20% of idiopathic PAH (IPAH) cases are caused by mutations in *BMPR2*^3^. Over the last 20 years, 20 further genes that increase the risk of PAH have been reported, which together contribute an additional ∼5% of PAH heritability. It is anticipated that the advent of multi-omic approaches can elucidate the aetiology of the remaining cases and explain the extent to which non-coding variation, variation at more than one locus, allelic and locus heterogeneity and common variation contribute to disease penetrance and expressivity.

In this review, we summarise the established and emerging knowledge about the genetic architecture of PAH, technological and statistical approaches to new risk gene discovery and discuss the clinical utility and therapeutic implications of genetic testing.

## State of the art

### Autosomal dominant forms of PAH

#### The TGF-*β* superfamily and PAH

In 2000, genetic analysis of families with PAH identified heterozygous germline mutations in *BMPR2*, the gene encoding bone morphogenetic protein receptor type 2, a member of the transforming growth factor-*β* superfamily^4^. Subsequently, *BMPR2* mutations were also identified in patients with IPAH^2^. To date, over 600 individual mutations in *BMPR2* have been identified in PAH patients. Mutation types include nonsense, frameshift, splice-site, missense, and copy number variants^5–9^.

BMPR2 is highly expressed on pulmonary vascular endothelium, where it couples with the type I receptor (ALK1) in response to the circulating bone morphogenic protein (BMP) ligands, BMP9 and BMP10, with endoglin (ENG) serving as a coreceptor^10^. High expression of *ALK1* and *ENG* in pulmonary endothelium contributes to the lung-specific phenotype of patients harbouring mutations in *BMPR2*.

In pulmonary artery endothelial cells (PAECs) *BMPR2* knockout leads to endothelial dysfunction characterised by increased permeability, heightened proliferation and enhanced apoptosis. The downstream signalling depends on canonical SMAD signalling but also other non-canonical pathways^11^.

The loss of BMPR2 signalling also impacts on the secretion of vasodilatory and inflammatory molecules by endothelial cells such as IL-6, IL-8, E-selectin and eNOS^12^, facilitates a transition from mitochondrial oxidative phosphorylation to glycolysis^13^ and promotes endothelial-to-mesenchymal transition^14^. Additionally, loss of *BMPR2* in other cell types like smooth muscle cells (PASMC), fibroblasts, immune cells, cardiomyocytes and hematopoietic stem cells may contribute to disease development.

PASMCs with *BMPR2* haploinsufficiency show a hyperproliferative phenotype due to the loss of antiproliferative Smad1/5 signalling^15^. Significantly worse right ventricle function indices in patients with *BMPR2* mutations can be, at least partially, explained by the impact of a loss of *BMPR2* function on cardiomyocyte metabolism.

In the transgenic mouse harbouring a heterozygous *Bmpr2* C-terminal truncating mutation (R899X), the overexpression of a dominant-negative mutant *Bmpr2* allele in cardiomyocytes led to the accumulation of long-chain fatty acids and failure to develop adaptive right ventricle hypertrophy^16^. There is mounting evidence that PAH is associated with myeloproliferative disorders^17^. Likewise, patients with PAH demonstrate abnormalities in the bone marrow and hematopoietic progenitor cells. It was previously demonstrated that low dose lipopolysaccharide caused PH in genetically susceptible mice^18^. A follow-up study showed that the hematopoietic stem cell compartment is involved in the susceptibility to PH in *Bmpr2*^+∕−^ mice and that those mice can be rescued by hematopoietic stem cell transplantation^19^.

It is now established that around 70–80% of familial PAH and 10–20% of IPAH cases are caused by mutations in *BMPR2*^20^. Interestingly, BMPR2 expression and signalling are also decreased in PAH patients without *BMPR2* germline mutations^21–23^, which suggests that impaired BMPR2 signalling might be a universal feature of PAH. This has been further supported by the identification of deleterious variants in the key members of the canonical BMPR2 signalling pathway.

PAH can co-occur with hereditary hemorrhagic telangiectasia (HHT), a disease characterized by arteriovenous malformations in the lung, brain, liver, skin, and mucus membranes which implicates *ACVRL1* (*ALK1)* and *ENG* mutations in the pathogenesis of PAH^24–26^. It is important to note that a much larger proportion of HHT patient will develop PH secondary to pulmonary arteriovenous fistulas^27^. In some instances, *ALK1* and *ENG* associated I/HPAH can occur without clinical features of HHT^25, 27, 28^ with the caveat that HHT is characterised by age-related penetrance with clinical manifestations developing over the lifetime.

Sequencing of genes encoding BMP receptor signalling intermediaries led to the identification of rare sequence variants in *SMAD1*, *SMAD4*^29^ and *SMAD9*^30^. The role of *SMAD9* variants in the pathogenesis of PAH was further confirmed in larger cohorts^8^. Moreover, exome sequencing of individuals without deleterious variants in *BMPR2,* but with more than one family member diagnosed with PAH revealed mutations in caveolin-1, encoded by *CAV1*, which participates in colocalisation of BMP receptors^31^. A *de novo* variant (c.473delC, p.P158Hfs*23) was also found in a patient with IPAH^31^. A separate study identified a third *CAV1* frameshift mutation (c.471delC, p.D157fs) in an adult patient with PAH^32^. All three variants are located in the terminal exome and escape nonsense-mediated decay.

This was corroborated by functional analysis that demonstrated retention of the mutant protein in endoplasmic reticulum and sequestration of the wild-type protein, which, together, lead to the impairment of caveolae assembly^33^. In lung endothelial and mesenchymal cells, caveolin 1 is essential for the regulation of SMAD signalling^34, 35^. The c.474delA *CAV1* mutation leads to hyperphosphorylation of SMAD1, SMAD5 and SMAD8, consequently resulting in a reduction of the anti-proliferative function of caveolin 1, thereby supporting SMAD gain of function as the underlying molecular mechanism of disease in patients with this *CAV1* variant^36^.

Also, a rare mutation in *CAV1* has been linked to lipodystrophy and PAH in a young child^37^. The results of animal studies further supported the role of *CAV1* in the pathogenesis of PH as *CAV1* deficient mice developed changes in pulmonary vasculature consistent with those seen in human PAH^38–40^.

Finally, the NIHR BioResource –Rare Diseases (NBR) study identified associations between rare deleterious variants in BMPR2 ligands, *GDF2* and *BMP10* and PAH^8, 41^. Further, *in vitro* analysis demonstrated that missense variants in *GDF2* led to impaired cellular processing and secretion of BMP9. PAH patients carrying these mutations had reduced plasma levels of BMP9 and reduced BMP activity. Interestingly, plasma BMP10 levels were also markedly reduced in these individuals. Although overall BMP9 and BMP10 levels did not differ between PAH patients and controls, a subset of PAH patients had markedly reduced plasma levels of BMP9 and BMP10 in the absence of *GDF2* mutations. These findings support therapeutic strategies to enhance BMP9 or BMP10 signalling in PAH^41^.

#### Channelopathies in PAH

Beyond TGF-*β* signalling, there is a growing body of evidence supporting the role of channelopathies in PAH. In 2013, six different mutations were identified in the *KCNK3* gene (Potassium Channel, Subfamily K, Member 3) in PAH patients. Heterozygous *KCNK3* mutations were found in sporadic and familial cases in which they segregated with the disease. Patch-clamp experiments demonstrated a loss of function in all identified mutations^42, 43^.

The role of *KCNK3* was further supported by a Spanish study, which found two *KCNK3* variants in three individuals. Importantly one individual was homozygous with a particularly aggressive disease diagnosed at birth^44^. Additionally, two more variants were identified in the US PAH cohort^45^. To date, ten different *KCNK3* mutations have been described in PAH patients^45–48^. The *Kcnk3*-mutated rat model recapitulates severe PAH phenotype reported in humans^49^. *KCNK3* has been identified as a druggable target^50^.

A rare deleterious variant in *ABCC8*, encoding the ATP binding cassette subfamily C member 8, was found in a patient with childhood-onset IPAH. Further screening of initial and validation cohort identified deleterious, heterozygous, missense variants in patients with IPAH, familial PAH, and PAH associated with congenital heart disease. Functional studies confirmed the decreased activity of the ATP-sensitive potassium channel, adding evidence to the theory that a subset of PAH patients might be mechanistically described as having potassium channelopathy^51^.

Further support for this hypothesis came from the NBR study in PAH, which reported an association between rare deleterious variants in *ATP13A3* and *AQP1* and PAH^8^. *ATP13A3* encodes for cation-transporting ATPase 13A3, a member of the P-type ATPase family of proteins, highly expressed in a variety of vascular cell types. Identified mutations clustered within the catalytic phosphorylation domain, which is likely to have a functional impact.

*ATP13A3* knockdown in PAECs reduces cell proliferation, predisposes to early apoptosis and increases permeability^52^. Still, ATP13A3 function and substrate remain elusive. There is some evidence that ATP13A3 transports polyamines^53^*. Atp13a3* mutant mice develop PH and show decreased polyamine concentration in lung tissue^54^. Conversely, elevated polyamine metabolism and polyamine content of the lung were found in monocrotaline PH model^55^; of note, the administration of a polyamine biosynthesis inhibitor prevented PH development^56^. In keeping with this elevated polyamine, plasma levels were found to be of prognostic significance in IPAH patients^57^.

*AQP1* encodes for plasma membrane water channel aquaporin 1, which is essential for the maintenance of vascular tone. In a mouse model of hypoxia-induced PH, GapmeR inhibitors targeting AQP1 reversed PH^58^ and *Aqp1*-null mice had impaired EC migration and angiogenesis^59^. In contrast, increased aquaporin 1 levels promote PASMC migration and proliferation via *β*-catenin and its targets^60^.

#### PAH as a disorder of transcriptional regulation

In a proportion of cases, with accompanying syndromic features and early-onset disease, PAH can be described as a disorder of transcriptional regulation. To date, two transcription factors have been implicated in PAH, namely TBX4 (T-Box Transcription Factor 4) and SOX17 (SRY-Box Transcription Factor 17).

TBX4 is a member of a conserved family of genes that share a common DNA-binding domain, the T-box, and encode for transcription factors involved in the regulation of developmental processes. TBX4 is expressed in lung, trachea, atria^61^ and hindlimb^62^ and was initially associated with small patella syndrome, characterised by hypoplasia or aplasia of the kneecap, ossification defects of the ischia and inferior pubic rami, as well as feet abnormalities^63^. Array comparative hybridisation and sequencing of a population of children with PAH and concurrent mental retardation and/or dysmorphic features implicated *TBX4* in the pathogenesis of PAH^64^. It is now recognised that mutations in *TBX4* are the second most common mutation found in pediatric-onset PAH^32^. Pathogenic TBX4 variants have also been reported in adult-onset PAH indicative of bimodal age distribution^8^.

*SOX17* encodes a member of the SOX (SRY-related HMG-box) family of transcription factors involved in the regulation of angiogenic processes, including arteriovenous differentiation and development of the lung microvasculature^65–67^. Rare deleterious variants in *SOX17* have been found in a large cohort of whole-genome sequenced I/HPAH patients characterised by young age at diagnosis, some of these variants also segregated with the phenotype^8^. These findings were validated in a Japanese cohort of I/HPAH patients^68^. Another study involving whole-exome sequencing of 256 patients implicated *SOX17* in the pathogenesis of PAH associated with congenital heart disease^69^.

### Autosomal recessive forms of PAH

Pulmonary veno-occlusive disease (PVOD) and capillary pulmonary hemangiomatosis (PCH) are subphenotypes of PAH group 1. They are histologically characterised by extensive venous and capillary involvement with only occasional pulmonary arterial changes. The clinical course of PVOD/PCH is also distinct with typically early-onset, rapidly progressive vasculopathy, low DLCO and lack of response to vasodilators. Biallelic (homozygous and compound heterozygous) *EIF2AK4* variants have been detected in hereditary and sporadic forms of PVOD^70^ and PCH^45^. Moreover, biallelic *EIF2AK4* variants were found in patients with a clinical diagnosis of PAH and conferred poor prognosis; the radiological assessment was unable to distinguish reliably between these patients and patients with idiopathic PAH^45, 71^.

These discoveries prompted the revision of the clinical classification of PAH and inclusion of PVOD/PCH as a subgroup of Group 1 PAH. *EIF2AK4,* also known as general control non-depressible 2 (GCN2), encodes a serine/threonine-protein kinase that phosphorylates the alpha subunit of eukaryotic initiation factor 2, which plays a key role in modulation amino acid metabolism in response to nutrient deprivation.

## The new kids on the block

The identification of additional genes harbouring potentially causal rare variants in PAH with smaller effect size requires a large collaborative effort to ensure adequate study power. Over the last few years, three consortia have developed large scale genomic and multi-omics programs to uncover the missing genetic architecture of PAH and to gain in-depth insight into disease pathobiology (The US PAH Biobank http://www.pahbiobank.org, NBR^8, 72^ and PVDOMICS^73^).

Through such efforts, the US PAH Biobank which consists of 37 US PAH centres, has recently identified two new PAH risk genes, tissue kallikrein 1 (*KLK1*) and gamma-glutamyl carboxylase (*GGCX*) and has confirmed many previously reported genes using a variable threshold method^9^. In a mixed cohort of patients with Group 1 PAH, a total of 12 cases harbouring *KLK1* variants (10 IPAH, 2 APAH) and 28 cases carried *GGCX* variants (17 IPAH, 9 APAH, 1 FPAH, 1 unknown subclass) were found.

The variants in *KLK1* included 1 stop-gain, 2 frameshifts, 1 splicing and 8 missense variants, whereas those in *GGCX* consisted of 5 nonsense, 1 frameshift, 21 missense and 1 in-frame deletion.

*KLK1* ^74^ is a component of the kallikrein-kinin system (KKS) implicated in the homeostasis of the cardiovascular, renal and central nervous system^75, 76^. The KKS comprises kallikreins, kininogens, kinins, kinin receptors and kininases (angiotensin-converting enzyme, ACE is the most important kinin-degrading enzyme in the cardiovascular system)^77^.

Fifteen tissue kallikreins have been discovered, of which only hK1 (encoded by *KLK1*) is a kininogenase, contributing to the formation of bradykinin (BK) and lys-bradykinin (lys-BK or kallidin), which exert their beneficial actions via nitric oxide and prostaglandins. In accordance with that animal and human studies found that BK B1 and B2 receptor antagonists administration results in an increase in systemic blood pressure^78–81^. Similarly, inactivating mutations in *Klk1* was also associated with systemic hypertension in spontaneously hypertensive rats^82^.

In humans, polymorphism in the regulatory region of *KLK1* is responsible for significant differences in hK1 expression and susceptibility to systemic hypertension^83, 84^. These findings suggested that KLK1 pathway might be a potential drug target; indeed, recombinant KLK1 treatment has been shown to improve the recovery in acute ischemic stroke by augmenting penumbral blood flow and suppressing inflammation^74^.

*GGCX*, on the other hand, encodes a protein which carboxylates glutamate residues of vitamin K-dependent proteins, a critical modification required for their activity. Vitamin K-dependent proteins impact many physiologic processes including coagulation, prevention of vascular calcification, and inflammation. Variants in *GGCX* have been previously associated with combined deficiency of vitamin K dependent clotting factors 1 and pseudoxanthoma elasticum-like disorder with multiple coagulation factor deficiency^85, 86^. Functional studies on the role of these genes in PH are still lacking.

A recent study by our group employed a novel Bayesian comparison method called BeviMed to discover new genotype-phenotype associations in a large cohort of deeply phenotyped patients with PAH on whom whole-genome sequencing was performed^87^. Using BeviMed, we identified another PAH candidate genes, *KDR* (kinase insert domain receptor), which encodes for vascular endothelial growth factor receptor 2 (VEGFR2)^87^.

We found that protein-truncating variants (PTV) in *KDR* were strongly associated with significantly reduced KCO and older age of onset. In addition to statistical evidence accompanied by biological plausibility, we also identified one case with a family history, which together with a recently published case report of two families, in which PTVs in *KDR* segregated with the phenotype of PAH and significantly reduced KCO^88^, amounts to three reported cases with familial segregation.

The role of VEGF signalling in the pathogenesis of PAH has been an area of intense research since reports of increased expression of VEGF, VEGFR1 and VEGFR2 in rat lung tissue in response to acute and chronic hypoxia^89^. An increase in lung VEGF has also been reported in rats with PH following monocrotaline exposure^90^. In humans, VEGF-A is highly expressed in plexiform lesions in patients with IPAH^91^, tracheal aspirates from neonates with a persistent PH of the newborn^92^ and small pulmonary arteries from infants with PH associated with a congenital diaphragmatic hernia^93^.

Given these findings, it is surprising that the overexpression of VEGFA ameliorates hypoxia-induced PAH^94^. In contrast, inhibition of VEGF signalling by SU5416 (sugen) combined with chronic hypoxia triggers severe angioproliferative PH^95^.

Sugen, a small-molecule inhibitor of the tyrosine kinase segment of VEGF receptors inhibits VEGFR1^96^ and VEGFR2^97^ causing endothelial cell apoptosis, loss of lung capillaries and emphysema^98^. In combination with chronic hypoxia, sugen causes cell-death dependent compensatory PAEC proliferation and severe PH^95^. Further evidence supporting the role of VEGF inhibition in the pathobiology of PAH comes from reports of PH in patients treated with bevacizumab^99^ and the multi-tyrosine kinase inhibitors^100, 101^.

## Factors influencing disease penetrance and expressivity

Penetrance is defined as the percentage of individuals with a particular mutation who exhibit a typical clinical phenotype. As such, penetrance is a measurement of the relationship between a genotype and phenotype. Understanding this relationship provides insight into the pathobiology of the disease and is fundamental for genetic counselling^102^.

Among well-established factors affecting penetrance are mutation type, individual variation in gene expression, epigenetic and environmental factors, as well as age and sex. Moreover, reduced penetrance may reflect the digenic or oligogenic inheritance. In PAH, female sex is the single most important determinant of the penetrance, which is estimated to be 42%, whereas penetrance in male carriers is 14%^103^. This is most likely driven by oestrogen metabolism^104–106^.

Variable gene expression was also found to impact on PAH penetrance; the expression levels of wild-type *BMPR2* from the unaffected allele transcript were higher in healthy carriers than in affected individuals^107^. Additionally, analysis of genetic variants affecting alternative splicing of BMPR2 showed that patients have a higher isoform B/A ratio than carriers^108^. Moreover, *in vitro* study on patient-specific induced pluripotent stem cells derived endothelial cells, it was shown that downregulation of endogenous receptor antagonists and upregulation of receptor activators might compensate for impaired BMPR2 signalling in unaffected *BMPR2* mutation carriers^109^. Somatic chromosomal abnormalities in lung tissue have been described as second-hit mutations affecting the BMPR2 pathway in PAH^110^

Related to penetrance is disease expressivity, which refers to a range and severity of signs and symptoms that occur in individuals with the same genetic condition. In PAH, it was shown that patients with missense mutations that escape nonsense-mediated decay have more severe disease than those with truncating mutations, suggestive of a dominant-negative impact of mutated protein on downstream signalling^111^. However, missense variants in the cytoplasmic tail appear to confer less severe phenotype than other *BMPR2* variants with a later age of onset, milder haemodynamics, and more vasoreactivity^112^.

Currently, the impact of environmental factors on PAH penetrance remains largely unknown, but studies show that BMPR2 expression and degradation can be affected by viral proteins and cocaine^113–115^.

## Gene-specific phenotypes

The clinical characterisation of patients harbouring rare deleterious variants in risk genes allows to understand better the functional impact of the mutation and in many instances has clinical and prognostic implications. Conversely, deep clinical phenotyping of patients with rare disorders permits clustering of those patients into homogenous cohorts, which may share common genetic architecture. To date, the best characterised group of mutation carriers are patients harbouring deleterious variants in *BMPR2.*

A large individual participant data meta-analysis has found that patients with *BMPR2* mutations have earlier disease onset, worse haemodynamics, are less likely to respond to NO challenge and have lower survival when compared to those without *BMPR2* mutations^116^. The histological analysis of lungs explanted from those patients also revealed a higher degree of bronchial artery hypertrophy/dilatation, which correlated with the frequency of haemoptysis at presentation^117^. Distinct phenotypes were also described in patients with rare deleterious variants in *TBX4*, *ENG*, *ALK1* and *EIF2AK4*.

Mutations in *TBX4*, known for its pivotal role in embryogenesis, present with severe PAH associated with bronchial and parenchymal changes, low DLCO, with or without skeletal abnormalities^118^, and bimodal age of onset^9^. Interestingly, the penetrance of *TBX4* mutations for skeletal abnormalities is much higher than for PAH^119^. Similarly, patients with variants in *ENG* and *ALK1* can present with signs and symptoms of either HHT, PAH or both^24, 120^.

Several studies have now shown that identification of biallelic mutations in *EIF2AK4* is sufficient to diagnose a hereditary form of pulmonary veno-occlusive disease even in the absence of typical radiographic features^121^. This is of importance as these patients present with rapidly progressive disease and may develop life-threatening pulmonary oedema in response to PAH medication. Detection of biallelic *EIF2AK4* mutation should trigger referral for lung transplantation^121^.

Recently, deep clinical phenotyping of patients with PAH combined with whole genome sequencing (WGS) revealed an association between protein-truncating *KDR* variants and PAH with reduced KCO and older age at diagnosis. Additionally, all patients were found to have mild interstitial lung disease. The frequency of systemic hypertension and thyroid dysfunction was also higher than in patients without the mutation in PAH risk genes^87^.

## Monogenic vs. digenic or oligogenic inheritance

HPAH has been historically considered a monogenic condition with an autosomal dominant mode of inheritance, meaning that a single deleterious variant in a PAH risk gene is sufficient to result in PAH phenotype. Nevertheless, 20 years of research into the genetic background of PAH indicates that none of the discovered genes is either sufficient or necessary for the disease to develop. Overall, low penetrance of PAH indicates that other environmental and genetic factors might be required for the disease to develop.

One of the possible explanations is that, at least in a proportion of cases, the inheritance is actually digenic or oligogenic. In the true digenic model, both genes are required to develop the disease. Conversely, in the composite class model, a variant in one gene is sufficient to produce the phenotype, but an additional variant in a second gene impacts the disease phenotype or alters the age of onset^122^. The latter model seems to be plausible in PAH, where co-occurrence of the variants in different PAH risk genes has been reported to impact on disease onset and penetrance^123^. Patients harbouring deleterious variants in more than one PAH risk gene were reported in small^124^ and large cohorts of HPAH patients^8^.

## COMMON GENETIC VARIATION

Although PAH is considered a Mendelian disorder, its low penetrance and heterogenous phenotype suggest a contribution of common sequence variation to disease susceptibility. In PAH several common genetic variants were found to contribute to phenotypic heterogeneity among patients with rare causal mutations in *BMPR2*. Polymorphisms at *BMPR2^125^ , TGF*-*β1^126^* and sex hormones loci^104^ were shown to contribute to variable gene expression and at least partially explain phenotypic variation between *BMPR2* mutation carriers.

In non-hereditary forms of PAH, a common polymorphism at the *CBLN2* (cerebellin) locus^127^ as well as in the endostatin^128^ and serotonin transporter genes^129^ were discovered. To date, the largest PAH GWAS study of multiple international I/HPAH cohorts identified two novel loci associated with PAH: an enhancer near *SOX17*, and a locus within *HLA-DPA1/DPB1* ^130^. These findings corroborate and extend the previous discovery of the association of rare variants in *SOX17* with PAH^8^.

One of the most successful applications of GWAS has been in the area of pharmacology. Pharmacogenetics aims to pinpoint DNA sequence variations that are associated with drug metabolism and efficacy as well as adverse effects. Recent studies have advanced our understanding of the influence of genetic variation on response to PAH therapy.

A study by Benza^131^ revealed that polymorphism in the endothelin-1 pathway was significantly associated with outcomes in patients treated with ERA. Likewise, common variants in *SIRT3* (mitochondrial deaminase) and *UCP3* (uncoupling protein 2), which regulates calcium entry to the cell, predicted response to dichloroacetate, pyruvate dehydrogenase kinase inhibitor in phase 2 clinical trial in PAH^132^. Concurrently genetic studies into mitochondrial DNA revealed that mitochondrial haplogroups influence the risk of PAH and that susceptibility to PAH emerged as a result of selective enrichment of specific haplogroups upon the migration of populations out of Africa^133^.

## Technological and statistical approaches to rare variant analysis

**Figure 1.**
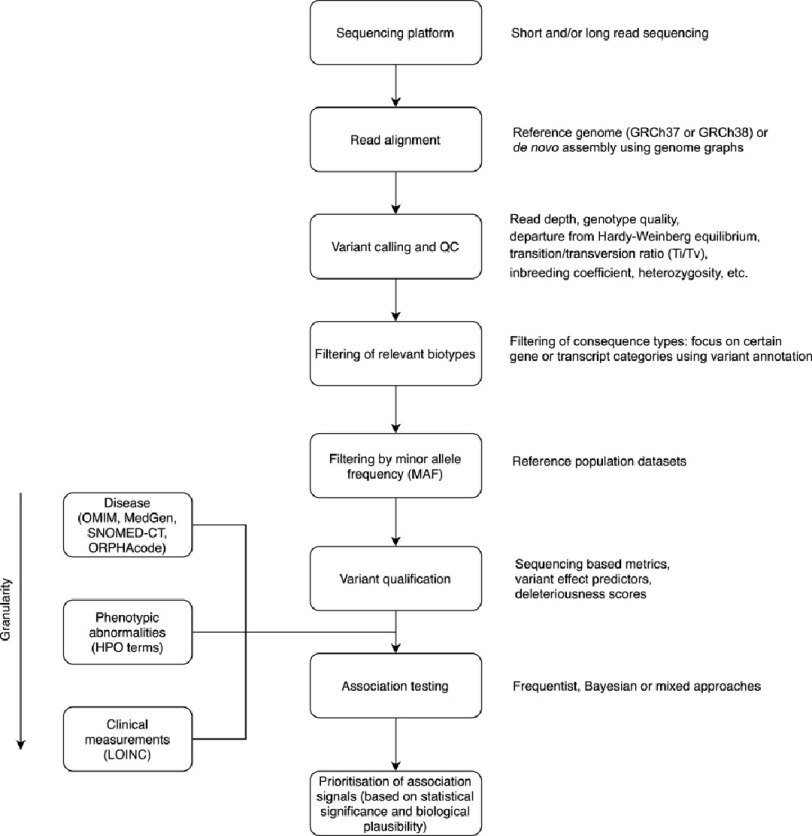
Common data processing and analysis pipeline for sequencing-based association studies. Abbreviations: QC, Quality Control; MAF, Minor Allele Frequency; HPO, Human Phenotype Ontology; CADD, Combined Annotation Dependent Depletion; LOINC, Logical Observation Identifiers, Names, and Codes; OMIM, Online Mendelian Inheritance in Man; SNOMED-CT, Systemized Nomenclature of Medicine—Clinical Terms; ORPHAcode, rare disease nomenclature maintained by Orphanet.

Rare variant analysis poses a number of challenges related to the sequencing, phenotyping, association testing and interpretation of rare variants. A common data processing and analysis pipeline for sequencing-based association studies is depicted in Figure 1.

### Sequencing

The development of ‘Sanger sequencing’ in the 1970s initiated a new era of human genomics; indeed, the advanced form of this technology remains the gold standard for sequencing^134^. Although highly accurate, Sanger sequencing proved to be costly and low throughput, and only the advent of “next-generation” sequencing (NGS) technology in the mid-2000s offered low-cost sequencing in both research and diagnostic settings.

Several sequencing modalities, such as targeted sequencing, whole-exome sequencing (WES) and low depth WGS, as well as sampling strategies (*i.e.* extreme phenotype sampling), have been developed to maximise resources and increase power.

WES, which enables sequencing of all protein-coding regions, gained traction, especially in Mendelian disorders. Although the protein-coding space occupies only 2% of the genome, it is estimated to harbour most of the disease causing variants^135, 136^. WGS enables analyses of coding and non-coding space, allowing for more comprehensive genomic testing. Rapid technological progress contributes to the variability in sequencing data acquired over time (differences in method and pipeline used, variable read lengths).

The additional biological variation that needs to be accounted for may come from the source of DNA (although saliva samples are a valid alternative to the blood samples, they contain a lower percentage of amplifiable DNA) or age at specimen collection. The latter can contribute to inflated rates of qualifying variants in older patients with age-associated clonal haematopoiesis^137–139^. Downstream analysis might be affected by genetic relatedness in the study cohort, unequal representation of ancestry groups^140^, male to female ratio in cases and controls^141, 142^ or choice of the transcript^143^.

### Phenotyping for genetics studies

No genetic study will lead to meaningful results without a comprehensive approach to characterising the phenotype of interest. Clinical diagnosis involves grouping patients based on observable traits, signs and symptoms, which are the product of genetic, epigenetic and environmental factors. As a result, clinical phenotypes can be dynamic and reactive, which is useful and desirable in the clinical setting but unsuitable for genetic studies.

There are many differences between clinical and research diagnosis (particularly diagnosis for genetic analyses) that need to be recognised and addressed. The former is obtained in three interrelated stages: history taking and physical examination, differential diagnosis and confirmation. These can be spread over time and involve multiple patient-practitioner encounters.

Research diagnosis is usually limited to a single encounter during which complete, reliable and valid phenotype description must be obtained. In research, setting completeness is assured by uniformed and standardised checklist (electronic case report forms, questionnaires) and reliability can be enhanced by the development of the standard operating procedure, diagnostic criteria for phenotypes, use of controlled vocabulary and regular staff training. Additionally, the phenotype description must be amenable to computational analysis. Finally, the validity of phenotypes is confirmed in test cohorts and through functional studies.

The precision with which the phenotype information is measured cannot be overestimated. In genetic studies mislabeling of participants or admixture of phenocopies can significantly affect power to detect an association^144^. Equally, categorising biologically continuous phenotypes (*i.e.* mPAP, DLCO) is prone to errors due to flaws in quantification methods and arbitrary thresholds.

Phenotype optimisation for genetic studies aims at finding homogenous groups of patients that likely share the same genetic architecture. This can be approached through various strategies: stratification based on family history, age of onset, sex, covariates-based methods which jointly estimate the effect of multiple variables and data reduction techniques. Alternatively, intermediate phenotypes (endophenotypes) can be used. Intermediate phenotypes are features closer to underlying biology that are at least as heritable as the phenotype itself, stable over time, and are associated with the disease of interest^145^.

Although clinical phenotyping remains the most widely used method of patients’ stratification both in clinical practice and research, it requires substantial domain knowledge and is very time-consuming. Deep computational phenotyping based on clinical and/or “omics” datasets using supervised or unsupervised machine learning might be an alternative due to unparalleled diagnostic precision and speed. At the heart of this approach are phenotype ontologies, like Human Phenotype Ontology, which allow standardised, highly granular and precise phenotyping across different disease domains^146^.

Use of ontologies to define phenotypes has already proven useful in identifying novel candidate genes for rare disorders^147^. Ontology-based analysis of phenotypes got further facilitated by the implementation of methods for manipulation, visualisation and computation of semantic similarity between ontological terms and sets of terms^148^. Complementary to this method is using a reverse phenotyping approach in which genetic marker data are used to infer about new phenotypes. This approach aims to cluster patients based on more deviant allele frequencies and validate findings in a separate sample or using resampling techniques^145^.

### Study design and statistical methods

Several recent publications have addressed the issue of study design and statistical methods in rare variant association analysis^149, 150^. As opposed to GWAS studies, single-variant testing is not suitable for rare variant analysis as the number of individuals carrying a particular variant might be very low and the effect size small requiring unrealistically large sample sizes. To circumvent this problem several gene- and region-based aggregation strategies have been proposed.

A gene-based aggregation approach uses gene boundaries to test for differences in the counts between cases and controls. This approach is useful when different variants confer an equivalent disease risk *i.e.* any loss-of-function variants (LOF; nonsense, frameshift, essential splice site) would result in the same phenotype. In such cases, the difference in presence or absence of LOF between cases and controls determines the association. Many variant filtering methods are routinely employed prior to association testing.

Firstly sequencing-based quality metrics, secondly MAF filters^151^ (usually MAF of 1:10 000 for autosomal dominant disorders and MAF of 1:1000 for autosomal recessive disorders), finally in silico deleteriousness scores for missense variants like PolyPhen-2^152^, SIFT^152, 153^, and REVEL^154^ and conservation scores like GERP^155^, PhyloP^156^ or PhastCons^157^, or the ensemble score CADD^158^ for genome-wide analysis, which combines several metrics in one score.

Given the known number of genes being tested (i.e., 20,000 protein-coding genes), the conventional adjustment for multiple testing within protein-coding space is calculated using Bonferroni formula, *α* = (0.05/20,000) ≈ 2.5 × 10^−6^. Importantly, if multiple models are applied, as is usually the case, the significance threshold needs to be further divided by the number of models tested.

Region-based collapsing approaches hinge on the notion that different regions within genes may vary in their tolerance to missense variation. An alternative approach, particularly useful in smaller studies, is collapsing variants that belong to the same gene set (i.e., genes that belong to the same pathway).

Complex genetic models like recessive or digenic/oligogenic mode of inheritance pose additional challenges. In the recessive mode of inheritance, MAF threshold must be relaxed as heterozygotes are unaffected (higher MAF in the reference populations); also, variants in *cis* configuration might be wrongly counted. Testing for digenic inheritance is particularly problematic due to the large number of possible combinations needing testing and adjusting for^150^.

A number of statistical methods have been developed to test for rare variant associations:

• Burden tests^159–161^ aggregate the information found within a predefined genetic region into a summary dose variable. In weighted burden tests^162^, variants are weighted according to their frequency or functional significance.
• Adaptive burden tests^163^ aim to account for bidirectional effects by selecting appropriate weights.
• Variance component (kernel) tests such as SKAT^164^ allow to test risk and protective variants simultaneously, but are underpowered when most variants are causal, and effects are unidirectional.
• Omnibus tests such as SKAT-O^165^, which combines burden test with the variance-component test, might be particularly useful when there is little knowledge of the underlying disease architecture.

In addition to frequentist approaches, Bayesian statistical framework offers a robust alternative. Bayesian model comparison methods, like BeviMed^166^, allow testing association between rare Mendelian disease and a genomic locus by comparing support for a model where disease risks depend on genotypes at rare variant sites in the locus and a genotype-independent “null” model.

The prior probability in such models can vary across variants (reflective of external biological information, *i.e.* depending on MAF, conservation scores, gene ontologies, expression in the tissue of interest) or be constant for all genes/variants reflecting the prior belief of the overall proportion of variants that are associated with a given phenotype. Population-based rare variant association studies can be complemented by family-based designs; these are particularly useful for dichotomous traits and robust to population stratification^167^.

Last but not least, an essential step in rare variant discovery is to ascertain the pathogenicity of a given variant and its causative role in the disease. Not all damaging variants are pathogenic and *in silico* approaches alone are not enough to predict if the variant is disease-causing^168^. To aid both research and clinical decision making, the American College of Medical Genetics and the Association for Molecular Pathology (ACMG) issued recommendations that combine and weight the computational, functional, population and clinical evidence to determine pathogenicity^169^. Other initiatives like ClinGen and ClinVar aim to define the clinical relevance of reported in the literature genes and variants for use in precision medicine and research^170^.

## Clinical utility and therapeutic implications of genetic testing in pah

### Clinical utility of genetic diagnosis

The utility of genetic diagnosis cannot be overestimated since it explains aetiology, informs prognosis and treatment decisions, and allows risk stratification of family members. Genetic testing has the potential to mitigate the disease course and has been recommended by current practice guidelines^171–174^. The influence of the mutation on outcomes has been well described in *BMPR2* and *EIF2AK4* mutation carriers. While *BMPR2* and *ACVRL1* mutation carriers present at a younger age, with more severe haemodynamics and worse survival than patients without pathogenic PAH mutations^3, 175, 176^, *EIF2AK4* mutation carriers present at a younger age than non-mutation carriers but the mutations status does not seem to affect prognosis^177^. Identification of *EIF2AK4* mutations allows confirming diagnosis not only in PVOD/PCH cases but also in patients who clinically presented as I/HPAH, eliminating the need to perform lung biopsy^178^. Due to dismal prognosis related to PVOD/PCH diagnosis, early referral for transplantation is warranted^121^.

### Pharmacogenetics

**Figure 2.**
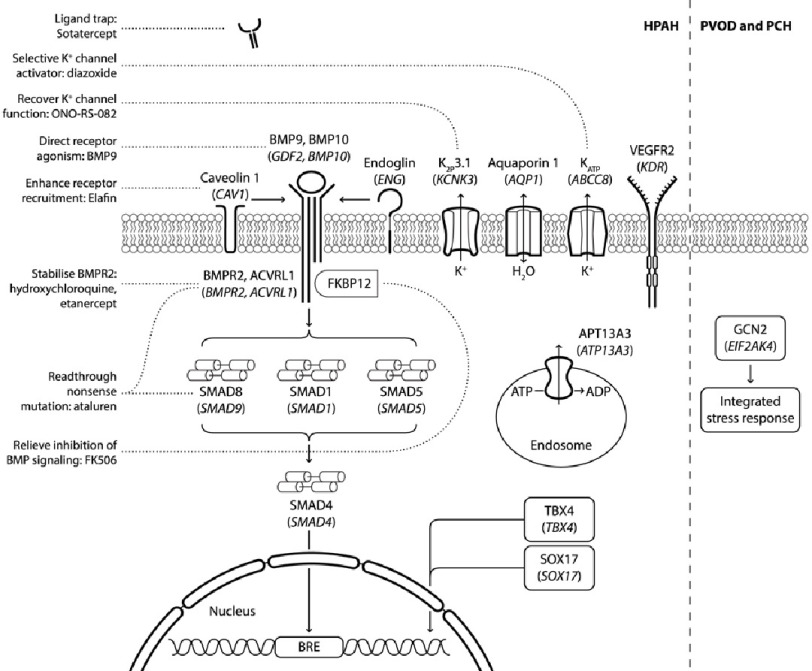
Druggable molecular pathways involved in the pathogenesis of PAH. Abbreviations: HPAH, heritable pulmonary arterial hypertension; BMP, bone morphogenetic protein, BMPR2, bone morphogenetic protein receptor type 2; ACVRL1 (also known as ALK1), activin receptor like 1; ENG, endoglin; CAV1, caveolin 1; KCNK3, Potassium Two Pore Domain Channel Subfamily K Member 3; ABCC8, ATP binding cassette subfamily C member 8; SOX17, SRY-Box Transcription Factor 17; TBX4, T-Box Transcription Factor 4; PVOD, pulmonary veno-occlusive disease; PCH, pulmonary capillary haemangiomatosis; ACVRL1, activin receptor- like 1; ATP13A3, cation-transporting ATPase13A3; BRE, BMP-responsive element; GCN2, general control nonderepressible 2; EIF2AK4, Eukaryotic Translation Initiation Factor 2 Alpha Kinase 4; FKBP12, 12kDa FK506-binding protein.

Knowledge about the genetic makeup of the individual allows targeted treatments enhancing efficacy and decreasing the risk of potential side effects^179^. Given the central role of BMP signalling in the pathogenesis of PAH, it is not surprising that therapies to enhance or rescue the BMPR2 pathway gained most of the traction (Figure 2).

In preclinical studies, BMP9 administration in heterozygous *BMPR2* knockout mice had a positive effect on haemodynamics, suggestive of a possibility to overcome *BMPR2* haploinsufficiency by up-titration of the ligand^10, 132^. Likewise, ataluren reads through nonsense mutations in *BMPR2* and *SMAD9* and restores the full-length proteins^180^. Chloroquine prevents progression of experimental PH through inhibition of lysosomal degradation of BMPR2, leading to increased receptor density at the surface of the endothelial cells^181^. Similarly, TNF- α inhibitor, etanercept, reduces inflammation, receptor shedding and proteasomal degradation of BMPR2^182^.

As reduced BMPR2 signalling is a known phenomenon even in patients without the mutation, targeting BMPR2 modifier genes might be an effective rescue mechanism. Enzastaurin was shown to rescue the *BMPR2* modifier gene, fragile histidine triad (*FHIT*) and reverse animal PH^183^. Ligand traps inhibiting negative BMPR2 regulation are another therapeutic option.

Recently Acceleron announced positive results from the sotatercept Phase 2 PULSAR trial in patients with PAH^184^. Sotatercept is a novel recombinant fusion protein that inhibits TGF-*β* superfamily members including GDF11 and activin A and B and restores the balance in the BMPR2 signalling pathway. Both EMA and FDA granted Sotatercept a breakthrough therapy status allowing expedited development process.

In the future, patients with deleterious variants in *CAV1*, may benefit from elafin, a peptidase inhibitor 3, encoded by *PI3* gene^185^. In sugen hypoxia rats, elafin reduced elastase activity and reversed PH, as well as improved endothelial function by increasing apelin^186^.

Tacrolimus (FK508), a potent immunosuppressant, was shown to increase BMP signalling via blocking FK-binding protein 12 in an animal PH model. The preclinical study confirmed the safety and tolerability of this agent in PAH^187^. Gremlin 1 secreted by vascular endothelium may inhibit BMPR2 signalling. Neutralizing antibodies interfering with gremlin 1 proved effective in ameliorating chronic hypoxia/sugen-induced PAH in mice^188^.

Besides the BMP pathway, modulating ion channels functions might be an effective therapeutic method. There is evidence that reduced potassium channel conductance in some *KCNK3* mutants can be recovered by the phospholipase A2 inhibitor ONO-RS-082^189^. Similarly, ABCC8 variants can be rescued by the SUR1 activator, diazoxide^51^.

### Knowledge, attitudes and barriers towards genetic testing and counselling

Despite recent developments in understanding the genetic background of PAH and the potential therapeutic implications, almost 80% of physicians caring for PAH patients never or rarely refer their patients to genetic counsellors or order genetic testing^190^. At least in the US, the most frequent reasons for that were reported to be lack of insurance coverage and limited access to genetic counsellors. Interestingly, the most important driver for genetic testing was patient inquiry^190^. This situation might be different in Europe where healthcare is publicly funded^191^, but it varies from country to country. In any case, a forward-thinking and innovative regulatory environment is necessary for integrating research and technological advances into clinical practice. In this respect, NHS England’s Genomic Medicine Service is likely to revolutionise routine patients’ care in the UK. Similar small- and large-scale initiatives are booming around the world. To give an example, the use of rapid WGS pioneered by Dr Stephen Kingsmore and his team at Rady Children’s Institute for Genomic Medicine enables precise diagnoses for critically-ill newborns within 26 hours^192^.

## Summary and future directions

In clinically diagnosed “idiopathic” PAH cohorts, up to 25% of patients have mutations in known PAH risk genes, leaving the remaining 75% without explanation about the disease trigger and pathobiology. Although environmental factors may account for some of these idiopathic cases, it seems likely that additional, unknown, rare genetic variation is responsible for many more cases. Moreover, both common and rare genetic factors may influence the penetrance of the known genes and disease expressivity. Multi-omic and spatial technologies offer an additional layer to the understanding of the disease pathobiology and are likely to enter clinical settings in the near future.

Only large-scale international collaborative efforts can collect the sample sizes powered to elucidate this missing heritability of PAH. Beyond that, pulmonary hypertension physicians’ education in genetics and genomics is crucial in the delivery of precision diagnostics and therapeutics. Access to genetic counselling and testing must be addressed at the national level and account for healthcare financing models and patients’ preferences.

## Funding sources

ES is a PhD student, founded by British Heart Foundation. SG is supported by the National Institute of Health Research (NIHR). NWM is a BHF Professor and an Emeritus NIHR Senior Investigator.

## References

[1] Newman JH (2005). Pulmonary hypertension. Am J Respir Crit Care Med.

[2] Thomson JR (2000). Sporadic primary pulmonary hypertension is associated with germline mutations of the gene encoding BMPR-II, a receptor member of the TGF-beta family [Internet]. Journal of Medical Genetics.

[3] Evans JDW, Girerd B, Montani D, Wang X-J, Galiè N, Austin ED (2016). BMPR2 mutations and survival in pulmonary arterial hypertension: an individual participant data meta-analysis. Lancet Respir Med.

[4] Lane KB, Machado RD, Pauciulo MW, Thomson JR, Phillips JA, Loyd JE (2000). Heterozygous germline mutations in BMPR2, encoding a TGF-*β* receptor, cause familial primary pulmonary hypertension [Internet]. Nature Genetics.

[5] Machado RD, Southgate L, Eichstaedt CA, Aldred MA, Austin ED, Best DH (2015). Pulmonary arterial hypertension: A current perspective on established and emerging molecular genetic defects. Hum Mutat.

[6] Machado RD, Eickelberg O, Elliott CG, Geraci MW, Hanaoka M, Loyd JE (2009). Genetics and genomics of pulmonary arterial hypertension. J Am Coll Cardiol.

[7] Machado RD, Aldred MA, James V, Harrison RE, Patel B, Schwalbe EC (2006). Mutations of the TGF-beta type II receptor BMPR2 in pulmonary arterial hypertension. Hum Mutat.

[8] Gräf S, Haimel M, Bleda M, Hadinnapola C, Southgate L, Li W (2018). Identification of rare sequence variation underlying heritable pulmonary arterial hypertension. Nat Commun.

[9] Zhu N, Pauciulo MW, Welch CL, Lutz KA, Coleman AW, Gonzaga-Jauregui C (2019). Novel risk genes and mechanisms implicated by exome sequencing of 2572 individuals with pulmonary arterial hypertension. Genome Med.

[10] Upton PD, Davies RJ, Trembath RC, Morrell NW (2009). Bone morphogenetic protein (BMP) and activin type II receptors balance BMP9 signals mediated by activin receptor-like kinase-1 in human pulmonary artery endothelial cells. J Biol Chem.

[11] Zhang YE (2017). Non-smad signaling pathways of the TGF- β family. Cold Spring Harb Perspect Biol [Internet].

[12] Gangopahyay A, Oran M, Bauer EM, Wertz JW, Comhair SA, Erzurum SC (2011). Bone morphogenetic protein receptor II is a novel mediator of endothelial nitric-oxide synthase activation. J Biol Chem.

[13] Caruso P, Dunmore BJ, Schlosser K, Schoors S, Dos Santos C, Perez-Iratxeta C (2017). Identification of MicroRNA-124 as a major regulator of enhanced endothelial cell glycolysis in pulmonary arterial hypertension via PTBP1 (Polypyrimidine Tract Binding Protein) and pyruvate kinase M2. Circulation.

[14] Ranchoux B, Antigny F, Rucker-Martin C, Hautefort A, Péchoux C, Bogaard HJ (2015). Endothelial-to-mesenchymal transition in pulmonary hypertension. Circulation.

[15] Yang X, Long L, Southwood M, Rudarakanchana N, Upton PD, Jeffery TK (2005). Dysfunctional smad signaling contributes to abnormal smooth muscle cell proliferation in familial pulmonary arterial hypertension [Internet]. Circulation Research.

[16] Hemnes AR, Brittain EL, Trammell AW, Fessel JP, Austin ED, Penner N (2014). Evidence for right ventricular lipotoxicity in heritable pulmonary arterial hypertension. Am J Respir Crit Care Med.

[17] Adir Y, Elia D, Harari S (2015). Pulmonary hypertension in patients with chronic myeloproliferative disorders [Internet]. European Respiratory Review.

[18] Soon E, Crosby A, Southwood M, Yang P, Tajsic T, Toshner M (2015). Bone morphogenetic protein receptor type II deficiency and increased inflammatory cytokine production. A gateway to pulmonary arterial hypertension. Am J Respir Crit Care Med.

[19] Crosby A, Toshner MR, Southwood MR, Soon E, Dunmore BJ, Groves E (2018). Hematopoietic stem cell transplantation alters susceptibility to pulmonary hypertension in Bmpr2-deficient mice. Pulm Circ.

[20] Evans JDW, Girerd B, Montani D, Wang X-J, Galiè N, Austin ED (2016). BMPR2 mutations and survival in pulmonary arterial hypertension: an individual participant data meta-analysis. Lancet Respir Med.

[21] Atkinson C, Stewart S, Upton PD, Machado R, Thomson JR, Trembath RC (2002). Primary pulmonary hypertension is associated with reduced pulmonary vascular expression of type II bone morphogenetic protein receptor. Circulation.

[22] Lavoie JR, Ormiston ML, Perez-Iratxeta C, Courtman DW, Jiang B, Ferrer E (2014). Proteomic analysis implicates translationally controlled tumor protein as a novel mediator of occlusive vascular remodeling in pulmonary arterial hypertension. Circulation.

[23] Chen N-Y, Collum SD, Luo F, Weng T, Le T-T, Hernandez AM (2016). Macrophage bone morphogenic protein receptor 2 depletion in idiopathic pulmonary fibrosis and Group III pulmonary hypertension. Am J Physiol Lung Cell Mol Physiol.

[24] Trembath RC, Thomson JR, Machado RD, Morgan NV, Atkinson C, Winship I (2001). Clinical and molecular genetic features of pulmonary hypertension in patients with hereditary hemorrhagic telangiectasia. N Engl J Med.

[25] Harrison RE, Berger R, Haworth SG, Tulloh R, Mache CJ, Morrell NW (2005). Transforming growth factor-beta receptor mutations and pulmonary arterial hypertension in childhood. Circulation.

[26] Chaouat A (2004). Endoglin germline mutation in a patient with hereditary haemorrhagic telangiectasia and dexfenfluramine associated pulmonary arterial hypertension [Internet]. Thorax.

[27] Harrison RE, Flanagan JA, Sankelo M, Abdalla SA, Rowell J, Machado RD (2003). Molecular and functional analysis identifies ALK-1 as the predominant cause of pulmonary hypertension related to hereditary haemorrhagic telangiectasia. J Med Genet.

[28] Fujiwara M, Yagi H, Matsuoka R, Akimoto K, Furutani M, Imamura S-I (2008). Implications of mutations of activin receptor-like kinase 1 gene (ALK1) in addition to bone morphogenetic protein receptor II gene (BMPR2) in children with pulmonary arterial hypertension. Circ J.

[29] Nasim MT, Ogo T, Ahmed M, Randall R, Chowdhury HM, Snape KM (2011). Molecular genetic characterization of SMAD signaling molecules in pulmonary arterial hypertension. Hum Mutat.

[30] Shintani M, Yagi H, Nakayama T, Saji T, Matsuoka R (2009). A new nonsense mutation of SMAD8 associated with pulmonary arterial hypertension. J Med Genet.

[31] Austin ED, Ma L, LeDuc C, Rosenzweig EB, Borczuk A, Phillips JA (2012). Whole exome sequencing to identify a novel gene (Caveolin-1) associated with human pulmonary arterial hypertension [Internet]. Circulation: Cardiovascular Genetics.

[32] Zhu N, Gonzaga-Jauregui C, Welch CL, Ma L, Qi H, King AK (2018). Exome sequencing in children with pulmonary arterial hypertension demonstrates differences compared with adults. Circ Genom Precis Med.

[33] Copeland CA, Han B, Tiwari A, Austin ED, Loyd JE, West JD (2017). A disease-associated frameshift mutation in caveolin-1 disrupts caveolae formation and function through introduction of a de novo ER retention signal [Internet]. Molecular Biology of the Cell.

[34] Nohe A, Keating E, Underhill TM, Knaus P, Petersen NO (2005). Dynamics and interaction of caveolin-1 isoforms with BMP-receptors. J Cell Sci.

[35] Saldanha S, Bragdon B, Moseychuk O, Bonor J, Dhurjati P, Nohe A (2013). Caveolae regulate Smad signaling as verified by novel imaging and system biology approaches. J Cell Physiol.

[36] Marsboom G, Chen Z, Yuan Y, Zhang Y, Tiruppathi C, Loyd JE (2017). Aberrant caveolin-1-mediated Smad signaling and proliferation identified by analysis of adenine 474 deletion mutation (c.474delA) in patient fibroblasts: a new perspective on the mechanism of pulmonary hypertension. Mol Biol Cell.

[37] Han B, Copeland CA, Kawano Y, Rosenzweig EB, Austin ED, Shahmirzadi L (2016). Characterization of a caveolin-1 mutation associated with both pulmonary arterial hypertension and congenital generalized lipodystrophy. Traffic.

[38] Drab M, Verkade P, Elger M, Kasper M, Lohn M, Lauterbach B (2001). Loss of caveolae, vascular dysfunction, and pulmonary defects in caveolin-1 gene-disrupted mice. Science.

[39] Zhao Y-Y, Liu Y, Stan R-V, Fan L, Gu Y, Dalton N (2002). Defects in caveolin-1 cause dilated cardiomyopathy and pulmonary hypertension in knockout mice. Proc Natl Acad Sci U S A.

[40] Maniatis NA, Shinin V, Schraufnagel DE, Okada S, Vogel SM, Malik AB (2008). Increased pulmonary vascular resistance and defective pulmonary artery filling in caveolin-1-/- mice. Am J Physiol Lung Cell Mol Physiol.

[41] Hodgson J, Swietlik EM, Salmon RM, Hadinnapola C, Nikolic I, Wharton J (2020). Characterization of mutations and levels of BMP9 and BMP10 in pulmonary arterial hypertension. Am J Respir Crit Care Med.

[42] Ma L, Roman-Campos D, Austin ED, Eyries M, Sampson KS, Soubrier F (2013). A novel channelopathy in pulmonary arterial hypertension. N Engl J Med.

[43] Bohnen MS, Roman-Campos D, Terrenoire C, Jnani J, Sampson KJ, Chung WK (2017). The Impact of heterozygous KCNK3 mutations associated with pulmonary arterial hypertension on channel function and pharmacological recovery [Internet]. Journal of the American Heart Association.

[44] Navas Tejedor P, Tenorio Castaño J, Palomino Doza J, Arias Lajara P, Gordo Trujillo G, López Meseguer M (2017). An homozygous mutation in KCNK3 is associated with an aggressive form of hereditary pulmonary arterial hypertension. Clin Genet.

[45] Best DH, Sumner KL, Smith BP, Damjanovich-Colmenares K, Nakayama I, Brown LM (2017). EIF2AK4 mutations in patients diagnosed with pulmonary arterial hypertension. Chest.

[46] Ma L, Roman-Campos D, Austin ED, Eyries M, Sampson KS, Soubrier F (2013). A novel channelopathy in pulmonary arterial hypertension. N Engl J Med.

[47] Navas Tejedor P, Tenorio Castaño J, Palomino Doza J, Arias Lajara P, Gordo Trujillo G, López Meseguer M (2017). An homozygous mutation in KCNK3 is associated with an aggressive form of hereditary pulmonary arterial hypertension. Clin Genet.

[48] Higasa K, Ogawa A, Terao C, Shimizu M, Kosugi S, Yamada R (2017). A burden of rare variants in BMPR2 and KCNK3 contributes to a risk of familial pulmonary arterial hypertension. BMC Pulm Med.

[49] Lambert M, Capuano V, Boet A, Tesson L, Bertero T, Nakhleh MK (2019). Characterization of Kcnk3 -Mutated Rat, a Novel Model of Pulmonary Hypertension [Internet]. Circulation Research.

[50] Antigny F, Hautefort A, Meloche J, Belacel-Ouari M, Manoury B, Rucker-Martin C (2016). Potassium channel subfamily K member 3 (KCNK3) contributes to the development of pulmonary arterial hypertension. Circulation.

[51] Bohnen MS, Ma L, Zhu N, Qi H, McClenaghan C, Gonzaga-Jauregui C (2018). Loss-of-function ABCC8 mutations in pulmonary arterial hypertension. Circ Genom Precis Med.

[52] Liu B, Haimel M, Bleda M, Li W, Gräf S, Upton PD (2018). S42 characterizing ATP13A3 loss of function in pulmonary arterial hypertension (PAH) [Internet]. Fundamental mechanisms of pulmonary arterial hypertension.

[53] Madan M, Patel A, Skruber K, Geerts D, Altomare DA, Iv OP (2016). ATP13A3 and caveolin-1 as potential biomarkers for difluoromethylornithine-based therapies in pancreatic cancers. Am J Cancer Res.

[54] Legchenko E, Liu B, West J, Vangheluwe P, Upton P, Morrell N (2020). Protein truncating mutations in ATP13A3 promote pulmonary arterial hypertension in mice [Internet]. 13.01 - Pulmonary hypertension.

[55] Hoet PHM, Nemery B (2000). Polyamines in the lung: polyamine uptake and polyamine-linked pathological or toxicological conditions [Internet]. American Journal of Physiology-Lung Cellular and Molecular Physiology.

[56] Olson JW, Atkinson JE, Hacker AD, Altiere RJ, Gillespie MN (1985). Suppression of polyamine biosynthesis prevents monocrotaline-induced pulmonary edema and arterial medial thickening. Toxicol Appl Pharmacol.

[57] Rhodes CJ, Ghataorhe P, Wharton J, Rue-Albrecht KC, Hadinnapola C, Watson G (2017). Plasma metabolomics implicates modified transfer RNAs and altered bioenergetics in the outcomes of pulmonary arterial hypertension. Circulation.

[58] Schuoler C, Haider TJ, Leuenberger C, Vogel J, Ostergaard L, Kwapiszewska G (2017). Aquaporin 1 controls the functional phenotype of pulmonary smooth muscle cells in hypoxia-induced pulmonary hypertension. Basic Res Cardiol.

[59] Saadoun S, Papadopoulos MC, Hara-Chikuma M, Verkman AS (2005). Impairment of angiogenesis and cell migration by targeted aquaporin-1 gene disruption. Nature.

[60] Yun X, Jiang H, Lai N, Wang J, Shimoda LA (2017). Aquaporin 1-mediated changes in pulmonary arterial smooth muscle cell migration and proliferation involve β-catenin. Am J Physiol Lung Cell Mol Physiol.

[61] Arora R, Metzger RJ, Papaioannou VE (2012). Multiple roles and interactions of Tbx4 and Tbx5 in development of the respiratory system. PLoS Genet.

[62] Glaser A, Arora R, Hoffmann S, Li L, Gretz N, Papaioannou VE (2014). Tbx4 interacts with the short stature homeobox gene Shox2 in limb development. Dev Dyn.

[63] Bongers EMHF, Duijf PHG, van Beersum SEM, Schoots J, Van Kampen A, Burckhardt A (2004). Mutations in the human TBX4 gene cause small patella syndrome. Am J Hum Genet.

[64] Kerstjens-Frederikse WS, Bongers EMHF, Roofthooft MTR, Leter EM, Douwes JM, Van Dijk A (2013). TBX4 mutations (small patella syndrome) are associated with childhood-onset pulmonary arterial hypertension. J Med Genet.

[65] Matsui T, Kanai-Azuma M, Hara K, Matoba S, Hiramatsu R, Kawakami H (2006). Redundant roles of Sox17 and Sox18 in postnatal angiogenesis in mice. J Cell Sci.

[66] Lange AW, Haitchi HM, LeCras TD, Sridharan A, Xu Y, Wert SE (2014). Sox17 is required for normal pulmonary vascular morphogenesis. Dev Biol.

[67] Corada M, Orsenigo F, Morini MF, Pitulescu ME, Bhat G, Nyqvist D (2013). Sox17 is indispensable for acquisition and maintenance of arterial identity. Nat Commun.

[68] Hiraide T, Kataoka M, Suzuki H, Aimi Y, Chiba T, Kanekura K (2018). SOX17 mutations in japanese patients with pulmonary arterial hypertension. Am J Respir Crit Care Med.

[69] Zhu N, Welch CL, Wang J, Allen PM, Gonzaga-Jauregui C, Ma L (2018). Rare variants in SOX17 are associated with pulmonary arterial hypertension with congenital heart disease. Genome Med.

[70] Eyries M, Montani D, Girerd B, Perret C, Leroy A, Lonjou C (2014). EIF2AK4 mutations cause pulmonary veno-occlusive disease, a recessive form of pulmonary hypertension. Nat Genet.

[71] Hadinnapola C, Bleda M, Haimel M, Screaton N, Swift A, Dorfmüller P (2017). Phenotypic characterization of mutation carriers in a large cohort of patients diagnosed clinically with pulmonary arterial hypertension. Circulation.

[72] Turro E, Astle WJ, Megy K, Gräf S, Greene D, Shamardina O (2020). Whole-genome sequencing of patients with rare diseases in a national health system. Nature [Internet].

[73] Hemnes A, PVDOMICS Study Group (2019). PVDOMICS: early clinical findings across the spectrum of pulmonary hypertension [Internet]. A105. Glory Days: the Latest Clinical Research in Pah.

[74] Alexander-Curtis M, Pauls R, Chao J, Volpi JJ, Bath PM, Verdoorn TA (2019). Human tissue kallikrein in the treatment of acute ischemic stroke. Ther Adv Neurol Disord.

[75] Devetzi M, Goulielmaki M, Khoury N, Spandidos D, Sotiropoulou G, Christodoulou I (2018). Genetically-modified stem cells in treatment of human diseases: Tissue kallikrein (KLK1)-based targeted therapy (Review) [Internet]. International Journal of Molecular Medicine.

[76] Stone OA, Richer C, Emanueli C, van Weel V, Quax PHA, Katare R (2009). Critical role of tissue kallikrein in vessel formation and maturation [Internet]. Arteriosclerosis, Thrombosis, and Vascular Biology.

[77] Campbell DJ (2001). The kallikrein-kinin system in humans. Clin Exp Pharmacol Physiol.

[78] Carbonell LF, Carretero OA, Stewart JM, Scicli AG (1988). Effect of a kinin antagonist on the acute antihypertensive activity of enalaprilat in severe hypertension. Hypertension.

[79] Madeddu P, Parpaglia PP, Demontis MP, Varoni MV, Fattaccio MC, Anania V (1995). Early blockade of bradykinin B2-receptors alters the adult cardiovascular phenotype in rats. Hypertension.

[80] Squire IB, O’Kane KP, Anderson N, Reid JL (2000). Bradykinin B (2) receptor antagonism attenuates blood pressure response to acute angiotensin-converting enzyme inhibition in normal men. Hypertension.

[81] Duka A, Duka I, Gao G, Shenouda S, Gavras I, Gavras H (2006). Role of bradykinin B1 and B2 receptors in normal blood pressure regulation. Am J Physiol Endocrinol Metab.

[82] Woodley-Miller C, Chao J, Chao L (1989). Restriction fragment length polymorphisms mapped in spontaneously hypertensive rats using kallikrein probes. J Hypertens.

[83] Yu H, Song Q, Freedman BI, Chao J, Chao L, Rich SS (2002). Association of the tissue kallikrein gene promoter with ESRD and hypertension. Kidney Int.

[84] Hua H, Zhou S, Liu Y, Wang Z, Wan C, Li H (2005). Relationship between the regulatory region polymorphism of human tissue kallikrein gene and essential hypertension. J Hum Hypertens.

[85] Li Q, Schurgers LJ, Smith ACM, Tsokos M, Uitto J, Cowen EW (2009). Co-existent pseudoxanthoma elasticum and vitamin K-dependent coagulation factor deficiency: compound heterozygosity for mutations in the GGCX gene. Am J Pathol.

[86] Napolitano M, Mariani G, Lapecorella M (2010). Hereditary combined deficiency of the vitamin K-dependent clotting factors. Orphanet J Rare Dis.

[87] Swietlik EM, Greene D, Zhu N, Megy K, Cogliano M, Rajaram S (2019). Reduced transfer coefficient of carbon monoxide in pulmonary arterial hypertension implicates rare protein-truncating variants in KDR [Internet]. bioRxiv.

[88] Eyries M, Montani D, Girerd B, Favrolt N, Riou M, Faivre L (2020). Familial pulmonary arterial hypertension by KDR heterozygous loss of function [Internet]. European Respiratory Journal.

[89] Tuder RM, Flook BE, Voelkel NF (1995). Increased gene expression for VEGF and the VEGF receptors KDR/Flk and Flt in lungs exposed to acute or to chronic hypoxia. Modulation of gene expression by nitric oxide. J Clin Invest.

[90] Cho YJ, Han JY, Lee SG, Jeon BT, Choi WS, Hwang YS (2009). Temporal changes of angiopoietins and Tie2 expression in rat lungs after monocrotaline-induced pulmonary hypertension. Comp Med.

[91] Tuder RM, Chacon M, Alger L, Wang J, Taraseviciene-Stewart L, Kasahara Y (2001). Expression of angiogenesis-related molecules in plexiform lesions in severe pulmonary hypertension: evidence for a process of disordered angiogenesis [Internet]. The Journal of Pathology.

[92] Lassus P, Turanlahti M, Heikkilä P, Andersson LC, Nupponen I, Sarnesto A (2001). Pulmonary vascular endothelial growth factor and Flt-1 in fetuses, in acute and chronic lung disease, and in persistent pulmonary hypertension of the newborn [Internet]. American Journal of Respiratory and Critical Care Medicine.

[93] Shehata SM, Mooi WJ, Okazaki T, El-Banna I, Sharma HS, Tibboel D (1999). Enhanced expression of vascular endothelial growth factor in lungs of newborn infants with congenital diaphragmatic hernia and pulmonary hypertension. Thorax.

[94] Partovian C, Adnot S, Raffestin B, Louzier V, Levame M, Mavier IM (2000). Adenovirus-mediated lung vascular endothelial growth factor overexpression protects against hypoxic pulmonary hypertension in rats. Am J Respir Cell Mol Biol.

[95] Taraseviciene-Stewart L, Kasahara Y, Alger L, Hirth P, Mc Mahon G, Waltenberger J (2001). Inhibition of the VEGF receptor 2 combined with chronic hypoxia causes cell death-dependent pulmonary endothelial cell proliferation and severe pulmonary hypertension. FASEB J.

[96] Itokawa T, Nokihara H, Nishioka Y, Sone S, Iwamoto Y, Yamada Y (2002). Antiangiogenic effect by SU5416 is partly attributable to inhibition of Flt-1 receptor signaling. Mol Cancer Ther.

[97] Fong TA, Shawver LK, Sun L, Tang C, App H, Powell TJ (1999). SU5416 is a potent and selective inhibitor of the vascular endothelial growth factor receptor (Flk-1/KDR) that inhibits tyrosine kinase catalysis, tumor vascularization, and growth of multiple tumor types. Cancer Res.

[98] Kasahara Y, Tuder RM, Taraseviciene-Stewart L, Le Cras TD, Abman S, Hirth PK (2000). Inhibition of VEGF receptors causes lung cell apoptosis and emphysema. J Clin Invest.

[99] Garcia AA, Hirte H, Fleming G, Yang D, Tsao-Wei DD, Roman L (2008). Phase II clinical trial of bevacizumab and low-dose metronomic oral cyclophosphamide in recurrent ovarian cancer: a trial of the california, chicago, and princess margaret hospital phase II consortia [Internet]. Journal of Clinical Oncology.

[100] Montani D, Bergot E, Günther S, Savale L, Bergeron A, Bourdin A (2012). Pulmonary arterial hypertension in patients treated by dasatinib [Internet]. Circulation.

[101] El-Dabh A, Acharya D (2019). EXPRESS: Pulmonary hypertension with dasatinib and other tyrosine kinase inhibitors. Pulm Circ.

[102] Cooper DN, Krawczak M, Polychronakos C, Tyler-Smith C, Kehrer-Sawatzki H (2013). Where genotype is not predictive of phenotype: towards an understanding of the molecular basis of reduced penetrance in human inherited disease [Internet]. Human Genetics.

[103] Larkin EK, Newman JH, Austin ED, Hemnes AR, Wheeler L, Robbins IM (2012). Longitudinal analysis casts doubt on the presence of genetic anticipation in heritable pulmonary arterial hypertension. Am J Respir Crit Care Med.

[104] Austin ED, Cogan JD, West JD, Hedges LK, Hamid R, Dawson EP (2009). Alterations in oestrogen metabolism: implications for higher penetrance of familial pulmonary arterial hypertension in females. Eur Respir J.

[105] Mair KM, Harvey KY, Henry AD, Hillyard DZ, Nilsen M, MacLean MR (2019). Obesity alters oestrogen metabolism and contributes to pulmonary arterial hypertension. Eur Respir J [Internet].

[106] Austin ED, Hamid R, Hemnes AR, Loyd JE, Blackwell T, Yu C (2012). BMPR2 expression is suppressed by signaling through the estrogen receptor. Biol Sex Differ.

[107] Hamid R, Cogan JD, Hedges LK, Austin E, Phillips 3rd JA, Newman JH (2009). Penetrance of pulmonary arterial hypertension is modulated by the expression of normal BMPR2 allele. Hum Mutat.

[108] Cogan J, Austin E, Hedges L, Womack B, West J, Loyd J (2012). Role of BMPR2 alternative splicing in heritable pulmonary arterial hypertension penetrance. Circulation.

[109] Gu M, Shao N-Y, Sa S, Li D, Termglinchan V, Ameen M (2017). Patient-specific iPSC-derived endothelial cells uncover pathways that protect against pulmonary hypertension in BMPR2 mutation carriers [Internet]. Cell Stem Cell.

[110] Aldred MA, Comhair SA, Varella-Garcia M, Asosingh K, Xu W, Noon GP (2010). Somatic chromosome abnormalities in the lungs of patients with pulmonary arterial hypertension [Internet]. American Journal of Respiratory and Critical Care Medicine.

[111] Austin ED, Phillips JA, Cogan JD, Hamid R, Yu C, Stanton KC (2009). Truncating and missense BMPR2 mutations differentially affect the severity of heritable pulmonary arterial hypertension [Internet]. Respiratory Research.

[112] Girerd B, Coulet F, Jaïs X, Eyries M, Van Der Bruggen C, De Man F (2015). Characteristics of pulmonary arterial hypertension in affected carriers of a mutation located in the cytoplasmic tail of bone morphogenetic protein receptor type 2. Chest.

[113] Huber LC, Brock M (2013). Faculty Opinions recommendation of Downregulation of bone morphogenetic protein receptor axis during HIV-1 and cocaine-mediated pulmonary smooth muscle hyperplasia: implications for HIV-related pulmonary arterial hypertension [Internet]. Faculty Opinions –Post-Publication Peer Review of the Biomedical Literature.

[114] Chinnappan M, Mohan A, Agarwal S, Dalvi P, Dhillon NK (2018). Network of MicroRNAs mediate translational repression of bone morphogenetic protein receptor-2: Involvement in HIV-associated pulmonary vascular remodeling [Internet]. Journal of the American Heart Association.

[115] Durrington HJ, Upton PD, Hoer S, Boname J, Dunmore BJ, Yang J (2010). Identification of a lysosomal pathway regulating degradation of the bone morphogenetic protein receptor type II. J Biol Chem.

[116] Evans JDW, Girerd B, Montani D, Wang X-J, Galiè N, Austin ED (2016). BMPR2 mutations and survival in pulmonary arterial hypertension: an individual participant data meta-analysis. Lancet Respir Med.

[117] Ghigna M-R, Guignabert C, Montani D, Girerd B, Jaïs X, Savale L (2016). BMPR2 mutation status influences bronchial vascular changes in pulmonary arterial hypertension. Eur Respir J.

[118] Thoré P, Girerd B, Jaïs X, Savale L, Ghigna M-R, Eyries M (2020). Phenotype and outcome of pulmonary arterial hypertension patients carrying a TBX4 mutation [Internet]. European Respiratory Journal.

[119] Kerstjens-Frederikse WS, Bongers EMHF, Roofthooft MTR, Leter EM, Douwes JM, Van Dijk A (2013). TBX4 mutations (small patella syndrome) are associated with childhood-onset pulmonary arterial hypertension. J Med Genet.

[120] Lyle MA, Fenstad ER, McGoon MD, Frantz RP, Krowka MJ, Kane GC (2016). Pulmonary hypertension in hereditary hemorrhagic telangiectasia. Chest.

[121] Hadinnapola C, Bleda M, Haimel M, Screaton N, Swift A, Dorfmüller P (2017). Phenotypic characterization of mutation carriers in a large cohort of patients diagnosed clinically with pulmonary arterial hypertension. Circulation.

[122] Gazzo A, Raimondi D, Daneels D, Moreau Y, Smits G, Van Dooren S (2017). Understanding mutational effects in digenic diseases. Nucleic Acids Res.

[123] Eichstaedt CA, Song J, Benjamin N, Harutyunova S, Fischer C, Grünig E (2016). EIF2AK4 mutation as “second hit” in hereditary pulmonary arterial hypertension. Respir Res.

[124] Pousada G, Baloira A, Valverde D (2016). Pulmonary arterial hypertension associated with several mutations. 43 Pulmonary Circulation and Pulmonary Vascular Diseases.

[125] Hamid R, Cogan JD, Hedges LK, Austin E, Phillips 3rd JA, Newman JH (2009). Penetrance of pulmonary arterial hypertension is modulated by the expression of normal BMPR2 allele. Hum Mutat.

[126] Phillips 3rd JA, Poling JS, Phillips CA, Stanton KC, Austin ED, Cogan JD (2008). Synergistic heterozygosity for TGFbeta1 SNPs and BMPR2 mutations modulates the age at diagnosis and penetrance of familial pulmonary arterial hypertension. Genet Med.

[127] Germain M, Eyries M, Montani D, Poirier O, Girerd B, Dorfmüller P (2013). Genome-wide association analysis identifies a susceptibility locus for pulmonary arterial hypertension. Nat Genet.

[128] Damico R, Kolb TM, Valera L, Wang L, Housten T, Tedford RJ (2015). Serum endostatin is a genetically determined predictor of survival in pulmonary arterial hypertension. Am J Respir Crit Care Med.

[129] Roberts KE, Fallon MB, Krowka MJ, Benza RL, Knowles JA, Badesch DB (2009). Serotonin transporter polymorphisms in patients with portopulmonary hypertension. Chest.

[130] Rhodes CJ, Batai K, Bleda M, Haimel M, Southgate L, Germain M (2019). Genetic determinants of risk in pulmonary arterial hypertension: international genome-wide association studies and meta-analysis. Lancet Respir Med.

[131] Benza RL, Gomberg-Maitland M, Demarco T, Frost AE, Torbicki A, Langleben D (2015). Endothelin-1 Pathway Polymorphisms and Outcomes in Pulmonary Arterial Hypertension. Am J Respir Crit Care Med.

[132] Michelakis ED, Gurtu V, Webster L, Barnes G, Watson G, Howard L (2017). Inhibition of pyruvate dehydrogenase kinase improves pulmonary arterial hypertension in genetically susceptible patients. Sci Transl Med [Internet].

[133] Farha S, Hu B, Comhair S, Zein J, Dweik R, Erzurum SC (2016). Mitochondrial haplogroups and risk of pulmonary arterial hypertension. PLoS One.

[134] Sanger F, Nicklen S, Coulson AR (1977). DNA sequencing with chain-terminating inhibitors. Proc Natl Acad Sci U S A.

[135] Majewski J, Schwartzentruber J, Lalonde E, Montpetit A, Jabado N (2011). What can exome sequencing do for you?. J Med Genet.

[136] Kuhlenbäumer G, Hullmann J, Appenzeller S (2011). Novel genomic techniques open new avenues in the analysis of monogenic disorders [Internet]. Human Mutation.

[137] Genovese G, Kähler AK, Handsaker RE, Lindberg J, Rose SA, Bakhoum SF (2014). Clonal hematopoiesis and blood-cancer risk inferred from blood DNA sequence. N Engl J Med.

[138] Buscarlet M, Provost S, Zada YF, Barhdadi A, Bourgoin V, Lépine G (2017). DNMT3A and TET2 dominate clonal hematopoiesis and demonstrate benign phenotypes and different genetic predispositions [Internet]. Blood.

[139] Xie M, Lu C, Wang J, McLellan MD, Johnson KJ, Wendl MC (2014). Age-related mutations associated with clonal hematopoietic expansion and malignancies. Nat Med.

[140] Petrovski S, Goldstein DB (2016). Unequal representation of genetic variation across ancestry groups creates healthcare inequality in the application of precision medicine. Genome Biol.

[141] Gao F, Chang D, Biddanda A, Ma L, Guo Y, Zhou Z (2015). XWAS: A software toolset for genetic data analysis and association studies of the X chromosome. J Hered.

[142] Hao M, Zhao X, Xu W (2020). Competing risk modeling and testing for X-chromosome genetic association [Internet]. Computational Statistics & Data Analysis.

[143] Cummings BB, Karczewski KJ, Kosmicki JA, Seaby EG, Watts NA, Singer-Berk M Transcript expression-aware annotation improves rare variant discovery and interpretation [Internet].

[144] Buyske S, Yang G, Matise TC, Gordon D (2009). When a case is not a case: effects of phenotype misclassification on power and sample size requirements for the transmission disequilibrium test with affected child trios [Internet]. Human Heredity.

[145] Schulze TG, McMahon FJ (2004). Defining the phenotype in human genetic studies: forward genetics and reverse phenotyping. Hum Hered.

[146] Köhler S, Carmody L, Vasilevsky N, Jacobsen JOB, Danis D, Gourdine J-P (2019). Expansion of the Human Phenotype Ontology (HPO) knowledge base and resources. Nucleic Acids Res.

[147] Köhler S, Schulz MH, Krawitz P, Bauer S, Dölken S, Ott CE (2009). Clinical diagnostics in human genetics with semantic similarity searches in ontologies. Am J Hum Genet.

[148] Greene D, Richardson S, Turro E (2017). ontologyX: a suite of R packages for working with ontological data. Bioinformatics.

[149] Lin W-Y (2016). Beyond rare-variant association testing: pinpointing rare causal variants in case-control sequencing study [Internet]. Scientific Reports.

[150] Povysil G, Petrovski S, Hostyk J, Aggarwal V, Allen AS, Goldstein DB (2019). Rare-variant collapsing analyses for complex traits: guidelines and applications. Nat Rev Genet.

[151] Petrovski S, Todd JL, Durheim MT, Wang Q, Chien JW, Kelly FL (2017). An exome sequencing study to assess the role of rare genetic variation in pulmonary fibrosis [Internet]. American Journal of Respiratory and Critical Care Medicine.

[152] Adzhubei IA, Schmidt S, Peshkin L, Ramensky VE, Gerasimova A, Bork P (2010). A method and server for predicting damaging missense mutations. Nat Methods.

[153] Sim N-L, Kumar P, Hu J, Henikoff S, Schneider G, Ng PC (2012). SIFT web server: predicting effects of amino acid substitutions on proteins. Nucleic Acids Res.

[154] Ioannidis NM, Rothstein JH, Pejaver V, Middha S, McDonnell SK, Baheti S (2016). REVEL: An ensemble method for predicting the pathogenicity of rare missense variants. Am J Hum Genet.

[155] Cooper GM, Stone EA, Asimenos G, Green ED, Batzoglou S, NISC Comparative Sequencing Program (2005). Distribution and intensity of constraint in mammalian genomic sequence. Genome Res.

[156] Pollard KS, Hubisz MJ, Rosenbloom KR, Siepel A (2010). Detection of nonneutral substitution rates on mammalian phylogenies. Genome Res.

[157] Siepel A, Bejerano G, Pedersen JS, Hinrichs AS, Hou M, Rosenbloom K (2005). Evolutionarily conserved elements in vertebrate, insect, worm, and yeast genomes. Genome Res.

[158] Kircher M, Witten DM, Jain P, O’Roak BJ, Cooper GM, Shendure J (2014). A general framework for estimating the relative pathogenicity of human genetic variants. Nat Genet.

[159] Asimit JL, Day-Williams AG, Morris AP, Zeggini E (2012). ARIEL and AMELIA: testing for an accumulation of rare variants using next-generation sequencing data. Hum Hered.

[160] Morris AP, Zeggini E (2010). An evaluation of statistical approaches to rare variant analysis in genetic association studies. Genet Epidemiol.

[161] Li B, Leal SM (2008). Methods for detecting associations with rare variants for common diseases: application to analysis of sequence data. Am J Hum Genet.

[162] Madsen BE, Browning SR (2009). A groupwise association test for rare mutations using a weighted sum statistic. PLoS Genet.

[163] Ionita-Laza I, Buxbaum JD, Laird NM, Lange C (2011). A new testing strategy to identify rare variants with either risk or protective effect on disease [Internet]. PLoS Genetics.

[164] Wu MC, Lee S, Cai T, Li Y, Boehnke M, Lin X (2011). Rare-variant association testing for sequencing data with the sequence kernel association test. Am J Hum Genet.

[165] Lee S, Emond MJ, Bamshad MJ, Barnes KC, Rieder MJ, Nickerson DA (2012). Optimal unified approach for rare-variant association testing with application to small-sample case-control whole-exome sequencing studies. Am J Hum Genet.

[166] Greene D, Richardson S, Turro E, NIHR Bio Resource (2017). A fast association test for identifying pathogenic variants involved in rare diseases. Am J Hum Genet.

[167] Ionita-Laza I, Lee S, Makarov V, Buxbaum JD, Lin X (2013). Family-based association tests for sequence data, and comparisons with population-based association tests. Eur J Hum Genet.

[168] Eilbeck K, Quinlan A, Yandell M (2017). Settling the score: variant prioritization and Mendelian disease. Nat Rev Genet.

[169] Richards S, Aziz N, Bale S, Bick D, Das S, Gastier-Foster J (2015). Standards and guidelines for the interpretation of sequence variants: a joint consensus recommendation of the American College of Medical Genetics and Genomics and the Association for Molecular Pathology. Genet Med.

[170] Rehm HL, Berg JS, Brooks LD, Bustamante CD, Evans JP, Landrum MJ (2015). ClinGen —the clinical genome resource [Internet]. New England Journal of Medicine.

[171] Leter EM, Boonstra AB, Postma FB, Gille JJP, Meijers-Heijboer EJ, Vonk Noordegraaf A (2011). Genetic counselling for pulmonary arterial hypertension: a matter of variable variability [Internet]. Netherlands Heart Journal.

[172] Morrell NW, Aldred MA, Chung WK, Elliott CG, Nichols WC, Soubrier F (2019). Genetics and genomics of pulmonary arterial hypertension. Eur Respir J [Internet].

[173] McGoon M, Gutterman D, Steen V, Barst R, McCrory DC, Fortin TA (2004). Screening, early detection, and diagnosis of pulmonary arterial hypertension [Internet]. Chest.

[174] Chung WK, Austin ED, Best DH, Brown LM, Elliott CG (2015). When to offer genetic testing for pulmonary arterial hypertension. Can J Cardiol.

[175] Girerd B, Montani D, Coulet F, Sztrymf B, Yaici A, Jaïs X (2010). Clinical outcomes of pulmonary arterial hypertension in patients carrying an ACVRL1 (ALK1) mutation. Am J Respir Crit Care Med.

[176] Sztrymf B, Coulet F, Girerd B, Yaici A, Jais X, Sitbon O (2008). Clinical outcomes of pulmonary arterial hypertension in carriers of BMPR2 mutation. Am J Respir Crit Care Med.

[177] Montani D, Girerd B, Jaïs X, Levy M, Amar D, Savale L (2017). Clinical phenotypes and outcomes of heritable and sporadic pulmonary veno-occlusive disease: a population-based study. Lancet Respir Med.

[178] Galiè N, Humbert M, Vachiery J-L, Gibbs S, Lang I, Torbicki A, Simonneau G, Peacock A, Vonk Noordegraaf A, Beghetti M, Ghofrani A, Sanchez MAG, Hansmann G, Klepetko W, Lancellotti P, Matucci M, McDonagh T, Pierard LA, Trindade PT, Zompatori M, Hoeper M (2015). 2015 ESC/ERS Guidelines for the diagnosis and treatment of pulmonary hypertension. The Joint Task Force for the Diagnosis and Treatment of Pulmonary Hypertension of the European Society of Cardiology (ESC) and the European Respiratory Society (ERS). Eur Respir J.

[179] Nelson MR, Tipney H, Painter JL, Shen J, Nicoletti P, Shen Y (2015). The support of human genetic evidence for approved drug indications. Nat Genet.

[180] Drake KM, Dunmore BJ, McNelly LN, Morrell NW, Aldred MA (2013). Correction of nonsense BMPR2 and SMAD9 mutations by ataluren in pulmonary arterial hypertension. Am J Respir Cell Mol Biol.

[181] Dunmore BJ, Drake KM, Upton PD, Toshner MR, Aldred MA, Morrell NW (2013). The lysosomal inhibitor, chloroquine, increases cell surface BMPR-II levels and restores BMP9 signalling in endothelial cells harbouring BMPR-II mutations. Hum Mol Genet.

[182] Hurst LA, Dunmore BJ, Long L, Crosby A, Al-Lamki R, Deighton J (2017). TNF α drives pulmonary arterial hypertension by suppressing the BMP type-II receptor and altering NOTCH signalling. Nat Commun.

[183] Dannewitz Prosseda S, Tian X, Kuramoto K, Boehm M, Sudheendra D, Miyagawa K (2019). FHIT, a Novel Modifier Gene in Pulmonary Arterial Hypertension. Am J Respir Crit Care Med.

[184] Badesch D, Gibbs S, Gomberg-Maitland M, Humbert M, Mclaughlin V, Preston I (2019). PULSAR: A phase 2, randomized, double-blind, placebo-controlled study to assess the efficacy and safety of sotatercept (ACE-011) when added to standard of care (SOC) for treatment of pulmonary arterial hypertension (PAH). Pulmonary hypertension.

[185] Molhuizen HOF, J PL, Weghuis O, van Kessel G, Schalkwijk J (1994). Assignment of the human gene encoding the epidermal serine proteinase inhibitor SKALP (PI3) to chromosome region 20q12 →q13 [Internet]. Cytogenetic and Genome Research.

[186] Nickel NP, Spiekerkoetter E, Gu M, Li CG, Li H, Kaschwich M (2015). Elafin reverses pulmonary hypertension via caveolin-1-dependent bone morphogenetic protein signaling. Am J Respir Crit Care Med.

[187] Spiekerkoetter E, Sung YK, Sudheendra D, Scott V, Del Rosario P, Bill M (2017). Randomised placebo-controlled safety and tolerability trial of FK506 (tacrolimus) for pulmonary arterial hypertension. Eur Respir J [Internet].

[188] Ciuclan L, Sheppard K, Dong L, Sutton D, Duggan N, Hussey M (2013). Treatment with anti-gremlin 1 antibody ameliorates chronic hypoxia/SU5416-induced pulmonary arterial hypertension in mice. Am J Pathol.

[189] Ma L, Roman-Campos D, Austin ED, Eyries M, Sampson KS, Soubrier F (2013). A novel channelopathy in pulmonary arterial hypertension. N Engl J Med.

[190] Jacher JE, Martin LJ, Chung WK, Loyd JE, Nichols WC (2017). Pulmonary arterial hypertension: Specialists’ knowledge, practices, and attitudes of genetic counseling and genetic testing in the USA [Internet]. Pulmonary Circulation.

[191] Girerd B, Montani D, Jaïs X, Eyries M, Yaici A, Sztrymf B (2016). Genetic counselling in a national referral centre for pulmonary hypertension [Internet]. European Respiratory Journal.

[192] Miller NA, Farrow EG, Gibson M, Willig LK, Twist G, Yoo B (2015). A 26-hour system of highly sensitive whole genome sequencing for emergency management of genetic diseases. Genome Med.

